# Safe Etching Route of Nb_2_SnC for the Synthesis of Two-Dimensional Nb_2_CT_x_ MXene: An Electrode Material with Improved Electrochemical Performance

**DOI:** 10.3390/ma16093488

**Published:** 2023-04-30

**Authors:** Karan Kishor Singh, Soorya Pushpan, Shadai Lugo Loredo, Andrea Cerdán-Pasarán, J. A. Hernández-Magallanes, K. C. Sanal

**Affiliations:** 1 Facultad de Ciencias Químicas, Universidad Autónoma de Nuevo León, San Nicolas de los Garza 66455, Nuevo León, Mexico; 2 Facultad de Ingeniería Mecánica y Eléctrica, Universidad Autónoma de Nuevo León, San Nicolas de los Garza 66455, Nuevo León, Mexico

**Keywords:** niobium carbide, two-dimensional nanostructures, XRD, supercapacitors, electrochemical

## Abstract

In this study, low-temperature synthesis of a Nb_2_SnC non-MAX phase was carried out via solid-state reaction, and a novel approach was introduced to synthesize 2D Nb_2_CT_x_ MXenes through selective etching of Sn from Nb_2_SnC using mild phosphoric acid. Our work provides valuable insights into the field of 2D MXenes and their potential for energy storage applications. Various techniques, including XRD, SEM, TEM, EDS, and XPS, were used to characterize the samples and determine their crystal structures and chemical compositions. SEM images revealed a two-dimensional layered structure of Nb_2_CT_x_, which is consistent with the expected morphology of MXenes. The synthesized Nb_2_CT_x_ showed a high specific capacitance of 502.97 Fg^−1^ at 1 Ag^−1^, demonstrating its potential for high-performance energy storage applications. The approach used in this study is low-cost and could lead to the development of new energy storage materials. Our study contributes to the field by introducing a unique method to synthesize 2D Nb_2_CT_x_ MXenes and highlights its potential for practical applications.

## 1. Introduction

Renewable energy resources are significant because they help us to diminish our dependence on fossil fuels. They are leading us to a sustainable future where we can live without the threat of climate change and pollution. Energy storage systems are combinations of procedures and techniques used to store energy that help to incorporate renewable energy sources into smart energy grids. There are many technologies used for energy storage, which can be classified based on the purpose for which energy is stored. Primarily, they are classified into two main methods: electrical energy storage and thermal energy storage, which is further divided into mechanical, chemical, and electrochemical energy. Among all energy storage technologies, electrochemical energy storage supercapacitors are better able to handle high power conversion rates than batteries. Another advantage of supercapacitors is that their charging times are nearly thousands of times faster than those of batteries with similar capacities [1]. In order to raise both the performance capability of batteries and the overall effectiveness of an energy storage system, supercapacitors have been used in conjunction with batteries [2]. In general, supercapacitors have been used in two major domains: high-power applications, where short-time power peaks are utilized by supercapacitors, to boost energy in hybrid vehicles, for instance, or to start heavy diesel engines; and low-power applications, where batteries can be more reliable, the most common examples of which are UPS and security installations [3].

Depending on the storage technique or cell structure, supercapacitors can be classified into three major categories: electric double-layer capacitors (EDLCs), hybrid supercapacitors, and pseudo-supercapacitors, the imaginary capacitors [4]. The EDLC supercapacitors use van der Waals interactions to store power in Helmholtz double layers on the phase terminal among the electrode’s layer and the electrolyte. In EDLC supercapacitors, energy is stored in a non-Faradic manner without any electron exchange or redox reaction. In general, activated carbon is utilized as an electrode material for EDLC supercapacitors, due to their large surface areas, such that they are utilizable for numerous applications [5]. Pseudo-capacitors are also known as Faradic supercapacitors, where the electrode materials undergo redox processes and act as intermediaries in the electron transfer process. Redox processes and the transport of electrons cause the pseudo-capacitance to form at the electrode surface [6]. The third type of supercapacitor combines EDLCs and pseudo-capacitors and is referred to as a hybrid supercapacitor. It performs better than the other two types of capacitors due to its high volumetric and gravimetric energy. It has a higher energy density because of the Faradic reaction that occurs on the negative electrode, although hybrid supercapacitors are currently just being investigated in laboratory conditions.

There are numerous materials that are appropriate for providing charge storage mechanisms. In general, carbon contains materials and various types of transition metal oxides. It has been known for pseudo-capacitive materials in aqueous electrolytes. Recent studies have shown that incorporating nanocellulose and its derived composites in supercapacitor electrodes can significantly enhance their performance [7]. The electrode materials for supercapacitors are categorized as carbon-based materials (e.g., activated carbon materials (ACs), graphene and carbon nanotubes (CNTs), transition metal oxides, and conducting polymers (e.g., PANI, polythiophene (PTh), and polypyrrole). Two-dimensional (2D) materials have shown promising potential for applications in various fields, such as electrochemical sensing [8], energy storage [9], and water purification [10], because they hold some exceptional properties, such as physical [11], mechanical [12], chemical, optical, and electrical properties [13]. Two-dimensional materials have drawn the attention of researchers working in the domains of nanotechnology, electrochemistry, and materials science. A variety of two-dimensional nanomaterials have been discovered over the past 20 years in addition to graphene [14], layered double hydroxides (LHDs) [15], transition metal dichalcogenides (TMDs) [16], transition metal oxides (TMOs) [17], black phosphorous (BP) [18], graphite carbon nitride (g-C_3_N_4_) [19], and hexagonal boron nitride (h-BN) [20]. Due to homogeneous layer formation, tremendous surface-to-volume ratios, strong affinity to water molecules, and high surface charges, two-dimensional nanomaterials possess outstanding properties, such as ample Young’s moduli, thermal conductivity, and electrical conductivity, along with adaptable band gaps. A breakthrough in 2D materials research was made in 2011; in addition to these stacked nanomaterials, a family of 2D transition metal carbides, carbo-nitrides, and nitrides called “MXene” were first reported by Gogotsi et al. [21].

MXenes are generally obtained by the selective etching of the layer ”A” metal from the precursor MAX phase compound with the general formula M_n+1_AX_n_, where M belongs to the family of transition metals, where A represents the element from the main group and X stands for carbon (C) or nitrogen (N), with *n* = 1, 2, 3 [22]. So far, there are about 70 MAX phases reported in the literature, but the number of MXenes that have been well established and studied is very limited. So far, different types of MXenes have been reported [23], including Ti_3_C_2_, Ti_2_C, (Ti_0.5_, Nb_0.5_) C, (V_0.5_, Cr_0.5_)_3_ C_2,_ Ti_3_CN, Ta_4_C_3,_ Nb_2_C, V_2_C [24], W_1.33_C [25], Nb_4_C_3_ [26], etc. 

MXenes possess remarkable electrochemical performance and other desirable properties, such as hydrophilicity, malleability, and two-dimensional structures with atomic-layer thicknesses and micrometer-scale lateral dimensions. These characteristics make them an excellent choice for electrode materials, which are crucial in enhancing the electrochemical performance of SCs. The design of MXene electrode materials, including their architecture, surface terminations, interlayer spacing, and composites, is a critical factor in determining the electrochemical performance of supercapacitors [27]. Previous studies have demonstrated the potential of various MXenes, including Ti_3_C_2_T_x_, V_2_CT_x_, and Nb_2_CT_x_, for use in supercapacitors, with excellent electrochemical performance reported in several cases. For instance, Dall’Agnese et al. [28] reported the use of a Ti_3_C_2_T_x_ MXene as an electrode material for a symmetric supercapacitor, which exhibited high capacitance and excellent cycling stability. Similarly, Sandhya et al. [29] synthesized a V_2_CT_x_ MXene via a facile wet-chemical method and demonstrated its use as an electrode material for an asymmetric supercapacitor, which showed high specific capacitance and energy density. Furthermore, by using in situ electrochemical Raman spectroscopy investigation, Hu et al. [30] studied the capacitance behavior of Ti_3_C_2_T_x_ using aqueous electrolytes and three different types of sulfate ions (H_2_SO_4_, (NH_4_)_2_ SO_4_, and MgSO_4_) and came to the conclusion that the Ti_3_C_2_T_x_ electrode outperformed the other two electrolytes in terms of supercapacitor performance in an acidic medium. Ghidiu et al. [31] reported for the first time the clay-like Ti_3_C_2_T_x_ materials as supercapacitor electrodes in acidic electrolyte, and the performance of these materials was found to be very promising, with volumetric capacitance up to 900 F cm^−3^ or 245 F g^−1^. Lukatskaya et al. [32] concluded that the electrochemical behavior of Ti_3_C_2_T_x_ in H_2_SO_4_ is predominantly pseudo-capacitive, with specific capacitance near to 230 F g^−1^. Apart from Ti_3_C_2_T_x_, other MXenes, such as V_2_CT_x_ [33], Mo_2_CT_x_ [34], Mo_1.33_TiC_2_T_x_ [35], and Nb_2_CT_x_ [36,37], have shown promising performance in supercapacitor and energy storage applications. Nb_2_CT_x_ is not more studied as compared to the Ti_3_C_2_T_x_ MXene, despite its having significant potential for many applications, such as biosensors [38] and energy storage [39]; most of the possible applications are still to be explored. Niobium-based MXenes are theoretically proved to be more stable than titanium-based MXenes [15]. So far, various methods have been reported for the synthesis of Nb-based MXenes (Nb_2_CT_x_ and Nb_4_C_3_T_x_), in which different acids and reaction conditions have been used. HF (hydrofluoric acid) is the most common acid used for synthesis of Nb_2_CT_x_ MXenes [40] under different etching times, such as 24 h, 48 h, and 96 h [41]. Apart from HF, a mixture of HCL (hydrochloric acid) and LiF (lithium fluoride) is also used to avoid the toxicity due to HF [42]. The Nb-based MXenes Nb_2_CT_x_ and Nb_4_C_3_T_x_ have proved potential in most applications, such as cancer nanomedicine [43], HER [16], EMI shielding [44], electrochemical sensors [40], and photocatalytic activities [45].

In this study, we investigated Nb_2_CT_x_, a supercapacitor electrode material, based on a two-dimensional nanostructure. While H_3_PO_4_ etching was used for the synthesis of Nb_2_CT_x_ MXenes, the solid-state reaction used to obtain the non-MAX phase Nb-Sn-C occurs at 1000 °C under the flow of nitrogen. XRD, SEM, FTIR, XPS, and TEM are some of the techniques that were used to describe the produced materials to examine their structural and morphological characteristics. By performing tests, such as galvanostatic charge–discharge (GCD) and cyclic voltammetry (CV) analyses, the electrochemical performance of the Nb_2_CT_x_-modified electrodes was examined. The Nb_2_CT_x_-modified electrodes displayed good capacitance performance, with a specific capacitance of 502.97 Fg^−1^ and a capacitance retention of 32.64% at a current density of 4.4 Ag^−1^. The findings of this study show that Nb_2_CT_x_ has promise as an electrode material for supercapacitors.

## 2. Materials and Methods

### 2.1. Materials

Nb (niobium powder, <45 µm, 99.7% metal basis), Sn (<125 µm, 99.8% metal), graphite powder (<30 µm), isopropyl alcohol (C_3_H_8_O), potassium hydroxide (KOH), acetylene black, and Nafion solution (binder) were purchased from Sigma Aldrich. The electrochemical characterizations were performed with three-electrode assembly, in which an Ag/AgCl electrode was used as the reference electrode, a platinum-wire electrode (purchased from Top Sky Technology China, Shenzhen, China) was used as an auxiliary electrode, and nickel foam on which the prepared sample was deposited was used as the working electrode. A mixture of distilled water (DI) and ethanol was used for the preparation of the solution and the cleaning of electrode materials throughout the experiment.

### 2.2. Synthesis of Nb_2_SnC and Nb_2_CT_x_ MXenes 

The Nb, Sn, and graphite powders were mixed at a molar ratio of 2:1.1:1 with a mortar and pestle and then ball-milled for 8 h using a Retsch PM 100 planetary ball mill with a 500 mL stainless steel jar and 10 mm-diameter stainless steel balls. The ball-to-powder weight ratio was 10:1, and the milling speed was set to 300 rpm. The resulting powder mixture was pressed into pellets with a size of 10 mm diameter and 1 mm thickness. The pressure exerted by the hydraulic press during compaction was 50 MPa, and each pellet weighed 1 g. The pellets were then heated in an atmospheric controlled tube furnace at 1000 °C for 8 h with nitrogen gas flowing through it. After cooling to ambient temperature, the pellets were manually ground into Nb_2_SnC powders and stored in a dry area.

As we know, the synthesis of graphene and black phosphorous [46] is performed by mechanical exfoliation, but this method is unfeasible for layers in the M_n+1_AX_n_ phase, due to the substantial metallic bonds among “M” and “A” elements. Among M-A and M-X bonds, the M-A bonds are chemically more active in comparison to the M-X bonds [47], and MXene can be synthesized by etching out the “A” element from the MAX phase with very strong acids, such as hydrofluoric acid (HF), lithium fluoride (LiF), or a mixture of both [48,49], though more commonly, fluoride-containing etchant [31,32] or heating is used [50,51]. 

**Figure 1:**
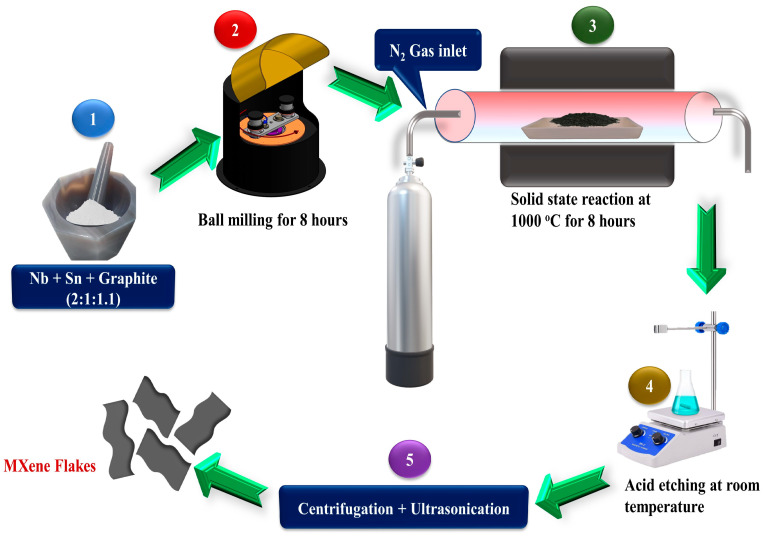
Schematic diagram for the synthesis of Nb2SnC non-MAX phase and Nb2CTx MXenes.

The use of hydrofluoric acid (HF) in the synthesis of MXenes has been considered challenging, time-consuming, and hazardous due to its toxic nature. In this work, we focused on developing a new approach for acquiring MXenes without using HF. To achieve this, 500 mg of Nb_2_SnC non-MAX phase powder was combined with 50 mL of phosphoric acid, and the mixture was magnetically swirled for 24 h at 60 °C. Following the 24-h period, the solution was washed using the same procedure as before and then dried for an additional 24 h at 70 °C in an oven. Overall, this method provides a safer and more feasible way to synthesize MXenes and can be a promising alternative to the traditional pathway involving HF as shown in schematic diagram Figure 1.

### 2.3. Structural and Morphological Characterizations

The arrangement of crystalline structures and phases present in the synthesized materials was identified using XRD with the Phillips Pan-Analytical X’-pert XRD system. The structural morphology of the synthesized sample was determined using SEM (scanning electron microscopy) with the Hitachi S-4800 at an applied potential of 2 kV. The elemental and atomic composition of the sample was calculated using EDS (energy-dispersive spectroscopy) with the Nova Nano 200 FEI Mark. XPS (X-ray photoelectron spectroscopy) was performed with the XPS Esca-lab 250Xi (Thermo Fisher Scientific, Waltham, MA, USA) instrument, which was used employing an 800 μm monochromatic Al-Kα-X-ray to analyze the sample’s surface chemistry as well as the electronic and chemical state of the element present in the prepared sample. The layered morphology and interlayer spacing were visible via HR-TEM using a JEM-2200FS microscope.

### 2.4. Preparation of Electrodes for Electrochemical Characterizations

A working electrode for three-electrode assembly was prepared by the drop-cast method. Homogeneous slurry was made by mixing 5 mg of etched Nb_2_CT_x_ MXenes with 25 μL of Nafion and 25 μL of ethyl alcohol. The solution was ultrasonicated for 3 h to make it homogeneous. After the sonication, the homogeneous solution was dropped on the nickel foam, which was washed with 2 M HCL prior to deposition several times until a uniform layer of material was obtained as an electrode. After the deposition, the nickel foam was dried at 70 °C overnight in the oven. 

The electrochemical characterizations were performed in a three-electrode assembly, and KOH was used as the electrolyte. The Ag/AgCl electrode and the platinum (Pt)-wire electrode were used as the reference and auxiliary electrode, respectively. Nickel foam surface modified with Nb_2_CT_x_ nanomaterial was used as the working electrode. The VMP3 multi-channel potentiostat electrochemical workstation was used for all electrochemical characterizations. The integral area of CV was used to determine the value of specific capacitance (F g^−1^):

(1) $$C_{s}=\frac{\int \mathrm{Idv}}{\delta \mathrm{Vm}}$$

where I is the current discharge, δ is the scan rate (mV s^−1^), V is the applied potential window, and m is the loading mass of the working electrode.

On the other hand, specific capacitance from the galvanostatic charge–discharge (GCD) curve was also calculated by finding out the integral area under the discharging curve using the following equation [52]:

(2) $$C_{s}=\frac{j_{s}\int_{t_{1}}^{t_{2}}Vdt}{\frac{V_{f\;}^{2}}{2}-\frac{V_{i\;}^{2}}{2}}$$

where $j_{s}$ is the current density, $\int_{t_{1}}^{t_{2}}Vdt$ is the area under the discharge curve, $V_{f}$ is the final potential, and $V_{i}$ is the initial potential during the GCD measurement.

## 3. Results and Discussion

### 3.1. Structural and Morphological Analysis

**Figure 2:**
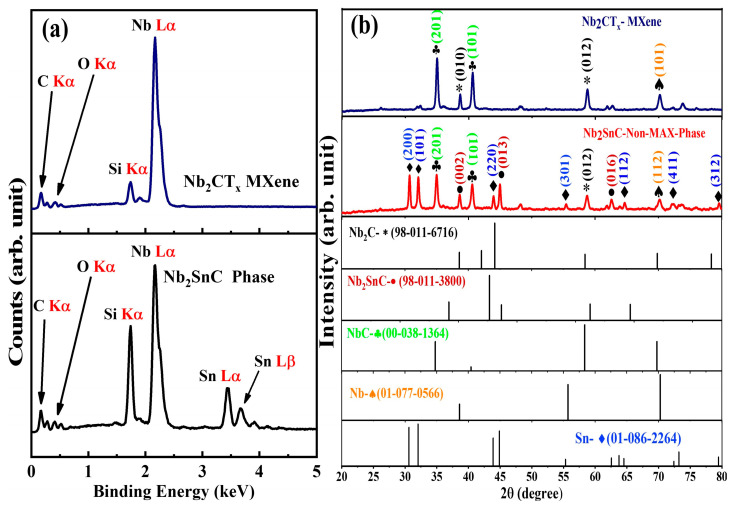
(**a**) EDS spectra of Nb_2_SnC and Nb_2_CT_x_ MXenes. (**b**) XRD patterns of Nb_2_SnC and Nb2CTx MXenes.

EDS analysis was used to identify the elemental composition of the Nb_2_SnC non-MAX phase and Nb_2_CT_x_ MXenes, as shown in Figure 2a. The reduction in the elemental composition of Sn (from 18.32% to 0.02%) and elevation in the elemental composition of C (from 7.72% to 54.37%) are evidence that Nb_2_CT_x_ MXenes were successfully formed. Additionally, compared to Nb_2_SnC, the elemental composition of Nb and C in the Nb_2_C MXenes was elevated. The oxygen present in the EDS spectra of the Nb_2_CT_x_ MXenes was associated with the intercalated water molecules and the surface terminations of OH ions. There were no impurities detected in the prepared sample. 

The XRDs of the Nb_2_SnC non-MAX phase and Nb_2_CT_x_ are displayed below in Figure 2b. As can be seen, the XRD analysis of Nb_2_SnC is consistent with the ICSD file (98-011-3800 hexagonal 63/mmc), with the planes (002), (013), and (016) found at the corresponding peaks 2θ = 38.76°, 45.01°, and 62.69°, respectively, as in the literature [53,54,55,56]. Additionally, the other peaks of Sn, Nb, NbC, and Nb_2_C are consistent with the corresponding ICSD files, 01-086-2264, 01-077-0566, 00-038-1364, and 98-011-6716, respectively. After the selective etching with H_3_PO_4_, the obtained Nb_2_CT_x_ MXene showed a similar pattern, with vanishing of the peaks at 2θ = 30.5°, 32.0°, 43.8°, 55.3°, 64.7°, and 72.25°, which belong to Sn. As can be seen in the XRD peaks, the Nb_2_SnC non-MAX phase has peaks with low intensity as compared with the MXene etched with Nb_2_CT_x_. The lattice parameters were calculated for the Nb_2_SnC NON-MAX phase and Nb_2_CT_x_. For hexagonal Nb_2_SnC, the lattice parameter was calculated as a = b = 2.90 Å and c = 12.9 Å, while for cubic Nb_2_CT_x_, the lattice parameter was calculated as a = 3.99 Å.

**Figure 3:**
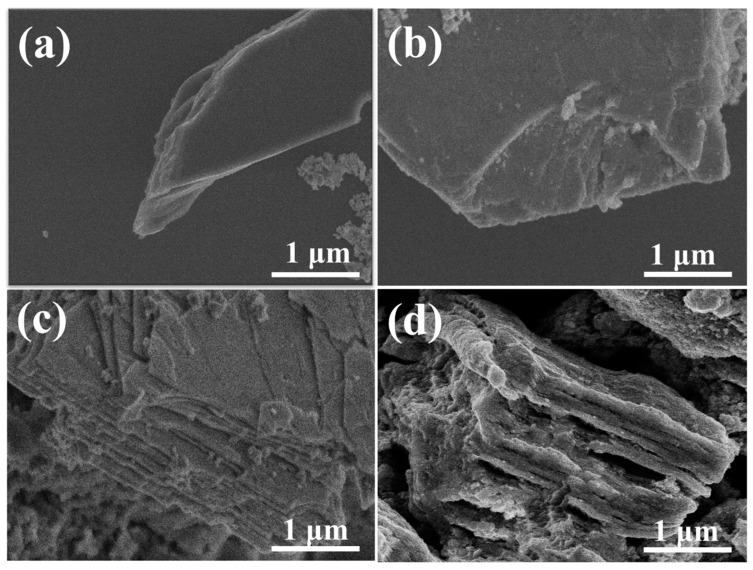
(**a**,**b**) SEM images of Nb_2_SnC NON-MAX phase. (**c**,**d**) SEM images of Nb_2_CT_x_ MXene.

The SEM images of the Nb_2_C MXene and the NON-MAX phase were analyzed to investigate surface morphology. The pure Nb_2_SnC bulk structure can be seen in Figure 3a,b. The morphology of the Nb_2_SnC non-MAX phase was altered to a sheet-like structure after being etched with phosphoric acid (H_3_PO_4_), as illustrated in Figure 3c,d. The morphology of the Nb_2_CT_x_ MXene is a structure that resembles two-dimensional sheets; the sizes of the layers’ structures vary, but they are consistently arranged. The space between the internal layers is expanded, which is more suitable for ion circulation and more convenient for the junction between active ions and the active sites of the material [57]. In another study in the literature [9], it was reported that nanoparticles with comparable elevated active surface areas could exhibit prominent electrochemical performance, and we could observe good surface areas in the Nb_2_CT_x_ materials, so these layered-structured nanomaterials are suitable for supercapacitor applications.

**Figure 4:**
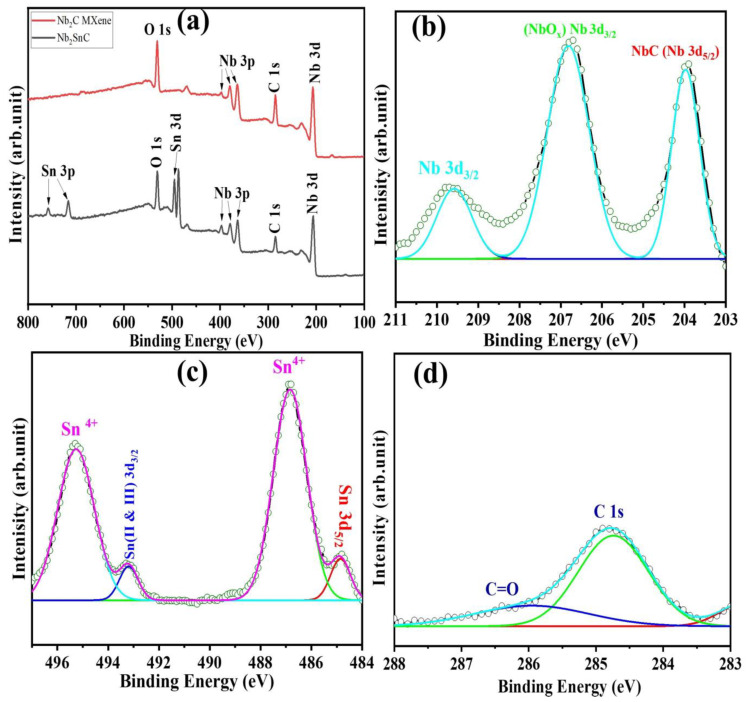
XPS high-resolution analysis of (**a**) Nb_2_SnC and Nb_2_CT_x_ survey spectra, (**b**) niobium (Nb), (**c**) tin (Sn), and (**d**) carbon (C).

XPS (X-ray photoelectron spectroscopy) was used to investigate the surface chemistry of the prepared sample along with the chemical state of the present elements with binding energy levels. Figure 4a show the XPS survey spectra of the Nb_2_SnC non-MAX phase and the Nb_2_CT_x_ MXene. Figure 4b shows the high-resolution spectrum of the Nb_2_CT_x_ MXene in the Nb 3d region, which could be best fitted with the corresponding Nb_2_C MXene (Nb 3d 204.8eV and Nb 3d_5/2_ 205.71 eV) and oxidized Nb (Nb 3d_5/2_ 209.72 eV) [58,59]. In Figure 4c, the peaks obtained at 496.3 eV and 487.65 eV are attributed to the binding energy of Sn4^+^, while those at 493.5 eV and 487.65 eV belong to that of metallic Sn [55,60]. In Figure 4d, the peaks of C 1s at 284.89 eV and 288.72 eV are ascribed to the binding energy of C-C and C=O bonds.

**Figure 5:**
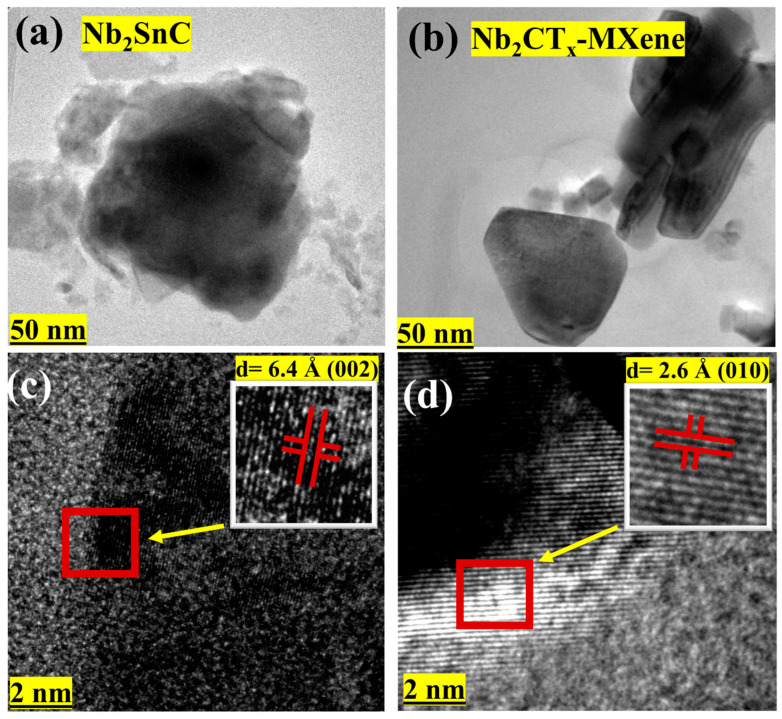
(**a**,**b**) TEM images of the Nb_2_SnC NON-MAX phase. (**c**,**d**) TEM images of the Nb_2_CT_x_ MXene.

To study the morphologies and structures of the prepared samples at atomic level, TEM analysis was performed. Figure 5a,b displays the TEM images of the Nb_2_SnC non-MAX phase at two different resolutions. The well layer structure of Nb_2_SnC NON-MAX can be seen in Figure 5a, which can also be confirmed from the SEM image of the Nb_2_SnC NON-MAX phase. For the same non-MAX phase, d-spacing calculated as shown in Figure 5b was found to be 6.4 Å, which corresponds to the (002) plane as compared to the XRD of the Nb_2_SnC non-MAX phase. Figure 5c,d display the TEM images of the Nb_2_CT_x_ MXene at two different resolutions. The two-dimensional layer of the Nb_2_CT_x_ MXene can be seen in Figure 5c at 50 nm resolution, which can also be confirmed from the SEM image of the same sample. The same sample d-spacing calculated as shown in Figure 5d was found to be 2.6 Å, which corresponds to the (010) plane as compared to the XRD of Nb_2_CT_x_.

### 3.2. Electrochemcial Analysis

Three-electrode assemblies were used for electrochemical determination for the Nb_2_CT_x_ MXene. For the investigation of electrochemical characteristics, cyclic voltammetry (CV), electrochemical impedance spectroscopy (EIS), and galvanostatic charge–discharge (GCD) analyses were performed. In a three-electrode assembly, nickel foam was used as a working electrode, modified by drop-casting of the sample on the nickel foam. 

**Figure 6:**
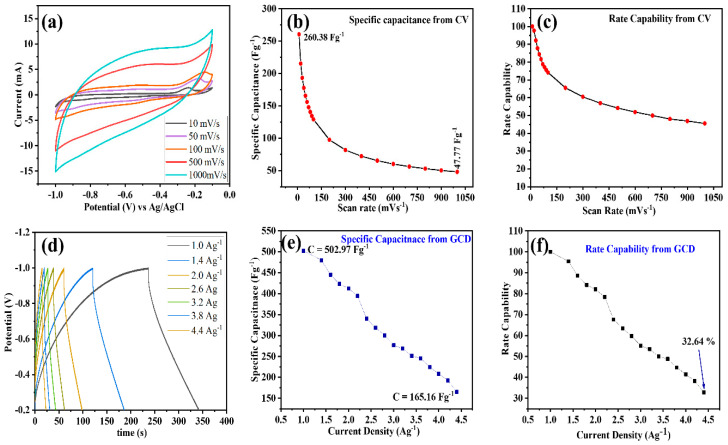
(**a**) CV for Nb_2_CT_x_ at various scan rates. (**b**) Specific capacitance vs. scan rate. (**c**) Rate capability calculated from CV. (**d**) GCD plot for Nb_2_CT_x_. (**e**) Specific capacitance vs. current density. (**f**) Rate capability calculated from GCD.

Cyclic voltammetry (CV) is a significant approach used to analyze the capacitive behavior and electrochemical performance of modified electrodes for supercapacitors. CV was run for the Nb_2_CT_x_ MXene, and the corresponding curves are shown in Figure 6a,b. All the CV curves were seen to have quasi-rectangular shapes, which suggest pseudo-capacitive behaviors [61]. In addition, the Nb_2_CT_x_ nanocomposite exhibits fragile and wide characteristics peaks, which is the outcome of oxidation–reduction reactions taking place at the surface of the electrode which demonstrate the pseudo-capacitive behavior of Nb_2_CT_x_. To further explicate the electrochemical performance of the Nb_2_CT_x_ nanocomposite, CV was performed at various scan rates, starting from 10 mVs^−1^ up to 1000 mVs^−1^ in the applied potential range from −1 V to −0.2 V, as shown in Figure 6a. Additionally, the CV curve exhibited a similar rectangular pattern up to a high scanning rate of 1000 mVs^−1^, which corresponds to adequate capacitance and rapid ion response. The specific capacitance at each scan rate was calculated from Equation (1), and these results are plotted in Figure 6b. 

The capacitance at 10 mVs^−1^ was found to be 260.38 Fg^−1^ and to exhibit a diminishing trend with stepwise increments in the scan rate, because, while increasing the scan rate, the diffusion of electrolyte ions into the internal electrode structure becomes challenging and there is no effective interaction between the electrode material and electrolyte, which leads to a decrease in specific capacitance. As the scan rate changed from 10 mVs^−1^ to 1000 mVs^−1^, the Nb_2_CT_x_ electrode retained the initial capacitance of 45.53% from its maximum value. The good rate capability may be elucidated by the high conductivity of the ions present in the electrolyte, which makes it appropriate for practical applications. This magnificent charge storage kinetic exhibits good electrochemical specifications, such as compact transfer resistance and smaller diffusion length [57].

The galvanostatic charge–discharge (GCD) technique is one of the electrochemical characterizations requisites for understanding the charging–discharging capability of a cell. For the Nb_2_CT_x_ MXene, GCD was performed at current densities ranging from 1.0 Ag^−1^ to 4.4 Ag^−1^ in the applied potential range between −0.2 V and −1.2 V to analyze the capacitance. The GCD curves at various current densities for the Nb_2_CT_x_ electrodes showed a symmetrical triangular pattern during the process of charging and discharging, which demonstrated EDLC behavior. The Nb_2_CT_x_ nanocomposite exhibited elongated charging and discharging durations, which correspond to the typical pseudo-capacitive behaviors of metal carbides and nitrides [61,62]. The specific capacitance value calculated from the GCD curve was found to be 502.97 Fg^−1^ for Nb_2_CT_x_ at the current density of 1.0 Ag^−1^, and it exhibited a decreasing trend up to 165 Fg^−1^ at the current density of 4.4 Ag^−1^, as shown in Figure 6d,e. Additionally, as the current density varied from 1.0 Ag^−1^ to 4.4 Ag^−1^, the Nb_2_CT_x_ nanocomposite electrode material retained 32.64% of its initial specific capacitance.

**Figure 7:**
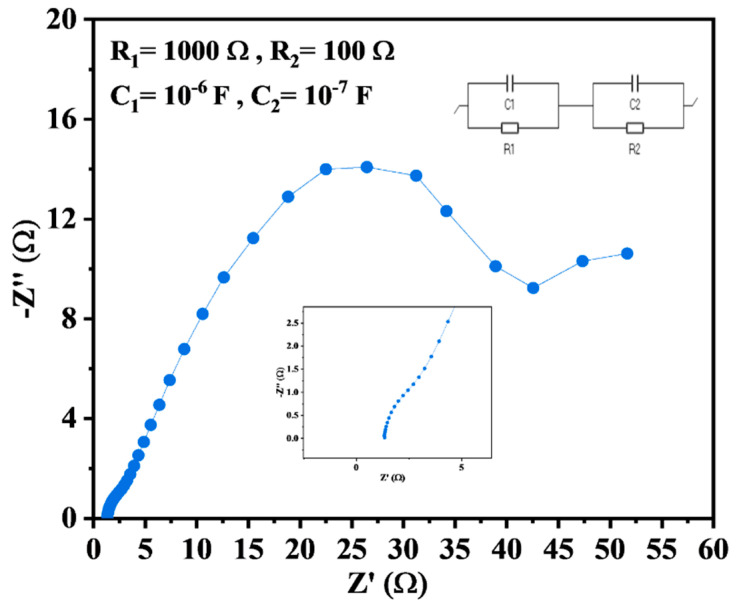
Nyquist plot for the Nb_2_CT_x_ MXene.

To further investigate the intrinsic resistance of the electrode and electrolyte, electrochemical impedance spectroscopy (EIS) was carried out at a frequency range of 100 MHz–100 KHz. Small electrode resistance was corroborated by EIS measurements, as shown in Figure 7, and the electrochemical performance of Nb_2_CT_x_ is attributed to favorable electrochemical reaction kinetics. The equivalent circuit was plotted along with the graph, and values of resistance and capacitance were calculated as mentioned in the graph. The equivalent series resistance was found to be 1.37 Ω. In the EIS curve, the linear behavior in the medium-frequency range can be attributed to the traditional capacitive behavior leading to EDLC behavior [63]. The superior electronic conductivity and charge-transfer kinetics of Nb_2_CT_x_ result in lesser charge transfer resistance, which helps in speeding up electrochemical reactions [64].

### 3.3. Analysis of the Supercapacitive Behavior of the 2D Nb_2_CT_x_ Nanomaterial

After analyzing all the electrochemical characterizations, the super capacitive behavior of the 2D Nb_2_CT_x_ nanomaterial was ascribed to the following aspects: (a) The sheet and layered morphology of the Nb_2_CT_x_ MXene, as shown in the SEM images, illustrates a prominent surface area and adequate conductivity, which reinforce the electrolytic diffusion and absorption of ions onto the electrode’s surface. (b) The presence of functional group -O in Nb_2_CT_x_, which was confirmed by EDS analysis after etching with phosphoric acid (H_3_PO_4_), helps in tuning the electrocatalytic properties, such as easy ion transfer, decreasing the internal resistance, and upgrading the electrical conductivity, which improves the electrochemical mechanism. The interlayer spacing in Nb_2_CT_x_ eases the way for fast hydrated ion diffusion, which affords kinetics similar to the EDLC behavior and accessible active sites to an extent which ensures high capacity and rate performance. A comparison table (Table 1) is provided below, after the literature review of some MXenes prepared under different reaction conditions and via different etching methods which have been reported for supercapacitors, which shows that the prepared Nb_2_CT_x_ MXene is a suitable candidate for supercapacitors.

## 4. Conclusions

- A novel synthesis method was developed for preparing Nb_2_SnC non-MAX phase powder at a lower temperature of 1000 °C, and two-dimensional nanostructures of Nb_2_CT_x_ MXenes were synthesized by selective etching of Sn-layered Nb_2_SnC using mild phosphoric acid (H_3_PO_4_).
- The hexagonal crystal structure of Nb_2_SnC and the cubic structure of Nb_2_CT_x_ were confirmed by analyzing the XRD patterns of the samples.
- During the formation of Nb_2_CT_x_ MXenes, the selective etching of Sn layers from Nb_2_SnC was evident in compositional analysis using EDX and XPS.
- Two-dimensional layered nanostructures of Nb_2_CT_x_ MXenes were observed in SEM images.
- The specific capacitance of the synthesized materials was evaluated using CV and GCD techniques. The CV plot of Nb_2_CT_x_ showed a specific capacitance of 260.38 Fg^−1^, while the GCD curve exhibited a specific capacitance of 502.97 Fg^−1^ for Nb_2_CT_x_.
- This study provides an eco-friendly and less hazardous method for synthesizing Nb_2_SnC and Nb_2_CT_x_. Nb_2_CT_x_ has superior electrochemical performance, making it a potential candidate for high-performance supercapacitor applications. The presented synthesis and characterization techniques could be useful for developing other MXenes and two-dimensional materials for energy storage applications.

## Figures and Tables

**Table 1:** Comparison of specific capacitances under various synthesis conditions.

MXene	Specific Capacitance	Etching Method	Scan Rate	Reference
Ti_3_C_2_T_x_	246 Fg^−1^	HCl + LiF 45 h	2 mVs^−1^	[31]
V_4_C_3_T_x_	209 Fg^−1^	50% HF 96 h	2 mVs^−1^	[65]
Nb_2_CT_x_/CNT	200 Fg^−1^	HCl + LiF 48 h	5 mVs^−1^	[42]
Nb_2_CT_x_	178 Fg^−1^	HCl + LiF 48 h	5 mVs^−1^	[42]
Nb_2_CT_x_	128 Fg^−1^	HF 48 h	2 mVs^−1^	[66]
Ti_3_C_2_/BCN	245 Fg^−1^	Etching/prolysis	2 mVs^−1^	[67]
Ti_3_C_2_T_x_ film	345 Fg^−1^	In situ etching	2 mVs^−1^	[68]
V_2_C	164 Fg^−1^	HF-free etching	5 mVs^−1^	[69]
Ti_3_C_2_T_x_/PPy	415 Fg^−1^	HCL + LiF 24 h	5 mVs^−1^	[70]
Nb_2_CT_x_	502.97 Fg^−1^	H_3_PO_4_ 24 h	10 mVs^−1^	This work

## Data Availability

Not applicable.
